# An experimental and theoretical investigation on the hyper-viscoelasticity of polyamide 12 produced by selective laser sintering

**DOI:** 10.1371/journal.pone.0304823

**Published:** 2024-07-29

**Authors:** Mahmoud Kadkhodaei, Marek Pawlikowski, Rafał Drobnicki, Janusz Domański

**Affiliations:** 1 Department of Mechanical Engineering, Isfahan University of Technology, Isfahan, Iran; 2 Institute of Mechanics and Printing, Warsaw University of Technology, Warszawa, Poland; UNIPI: Universita degli Studi di Pisa, ITALY

## Abstract

Polyamide 12 (PA12) is vastly utilized in many additive manufacturing methods, such as Selective Laser Sintering (SLS), and a better understanding of its mechanical behaviors promotes available knowledge on the behaviors of 3D-printed parts made from this polymer. In this paper, SLS-produced standard tensile specimens are studied under monotonic and cyclic tension tests, as well as stress relaxation experiments, and the obtained force-displacement responses are shown to be consistent with a hyper-viscoelastic material model. This finding is also observed in typical pantographic structures produced by the same manufacturing parameters. To propose a constitutive model for predicting these behaviors, the convolution integral of a strain-dependent function and a time-dependent function is developed where the material parameters are determined with the use of both short-term and long-term responses of the specimens. Numerical results of the presented model for standard test specimens are shown to be in good agreements with the experimental ones under various loading conditions. To prove the capabilities of the proposed model in studying any SLS-produced part, finite element implementation of the constitutive equations is shown to provide numerical results in agreement with the empirical findings for tensile loading of the 3D-printed pantographic structure.

## Introduction

Polyamide 12 (PA12 or Nylon-12) in powder form is one of the primary materials in additive manufacturing techniques, especially Selective Laser Sintering (SLS), because an appropriate difference between the crystallization and the melting temperatures of this polymer makes a relatively large processing window [1, 2] for 3D printing. Besides applications in industrial [3, 4] and medical areas [5–7], 3D-printed polyamide products can be employed in making sacrificial patterns for investment casting [8]. One of the hot topics on the investigation of SLS-produced PA12 has been their application in pantographic lattice structures, which are constructed from intersecting ‎arrays of parallel fibers. The arrays are interconnected by pivots or short rods so that ‎the whole configuration makes pantographs enable of undergoing very large deformations [9, 10]. Since the responses of pantographic structures can be tuned by adjustments in their topology and geometry, they have been considered mathematically-driven metamaterials in many theoretical studies [11–13].

Modeling and numerical simulation of mechanical responses for SLS-produced PA12 parts have been mostly performed for pantographic structures under various types of monotonic loadings [14], and a number of topics has been investigated by usually considering a linear elastic base material [15–19]. However, characterizations of polyamide 12 have revealed several kinds of material nonlinearity in the mechanical behaviors of this polymer [20, 21]. Taş [22] investigated simple tension test of SLS-made PA12 specimens and presented an elastoplastic model to describe the experimental responses. He employed isotropic hardening, supported with rate dependent yielding by using Cowper-Symonds (overstress power) law, in his approach. Lindberg [23] also presented an elastoplastic model for his products, which were found to be anisotropic. Thus, he utilized a linear elastic transversely isotropic material model where Hill’s yield criterion was used. Sagradov et al. [24] studied simple tension, as well as multi-step relaxation, tests and examined different approaches to present a model for the observed responses. For the application of the investigated material models without coupled damage, they found Chaboche model more suitable for tensile and multi tensile loading paths. On the other hand, numerical results of Bodner-Partom model showed a higher degree of correlation with the findings of experiments for relaxation and multi relaxation. A coupling with Lemaitre’s damage model showed an increase in the accuracy of numerical simulations only for tension and multi-tension tests coupled with the Chaboche material model.

Lammens et al. [25] performed uniaxial tension, compression, simple shear, and relaxation experiments and realized that time dependency appears in the mechanical responses of SLS-produced PA12. They suggested that viscoelastoplasticity is required to model their observed behaviors. Schneider and Kumar [26] investigated tensile, compressive, 3-point bending, shear and fracture tests on their SLS-made PA12 specimens. With the use of an available rheological model [27], they proposed a hyperelastic-viscoplastic approach to describe their experimental findings. Krönert et al. [28] conducted creep tests under different loads and analyzed creep behavior using the Burgers viscoelastic model. Time dependency causes the mechanical responses to vary cycle by cycle under cyclic loadings, and these details need to be studied for repetitive loadings. However, to the authors’ knowledge, most of the available works are concentrated on monotonic loadings, and a few investigations have been done on fatigue loading of polyamide 12 processed by selective laser sintering. Munguia et al. experimentally studied fatigue of laser-sintered PA12 specimens under four-point rotating ‎bending [29] and reversed bending [30]. Following these works, studies on theoretical estimation of fatigue lifetime were conducted. Santonocito [31] employed the so-called self-heating approach to evaluate fatigue life under uniaxial tensile loading. Salazar et al. [32] utilized the damage and fracture mechanics theories to study tensile fatigue loading on additively manufactured polyamide 12.

In this paper, standard tensile specimens are made by SLS, and their force-displacement curves are obtained under monotonic and cyclic tension tests under different strain rates, as well as stress relaxation experiments. The results reveal hyper-viscoelasticity in both short-term and long-term responses of the specimens, and this hypothesis is confirmed by conducting experiments on typical pantographic structures produced by the same 3D printing settings. To address the observed phenomena, a constitutive model is proposed founded on the convolution integral of a strain-dependent function and a time-dependent function. The model parameters identification is performed by means of curve-fitting procedure using the Levenberg-Marquardt algorithm whose input data are provided by both short-term and long-term responses of the specimens under different strain rates. Numerically-predicted results of the developed model for standard test specimens are shown to be in good agreements with the experimental findings under various loading conditions. Finite element implementation of the constitutive equations is conducted in software ANSYS, and the produced pantographic structure is simulated under uniaxial tension. This case study proves the capabilities of the proposed model in studying any SLS-produced part since the numerical results coincide with the empirical findings for tensile loading of the 3D-printed pantographic structure with a reasonable accuracy.

## Experimental specimens

**Fig 1:**
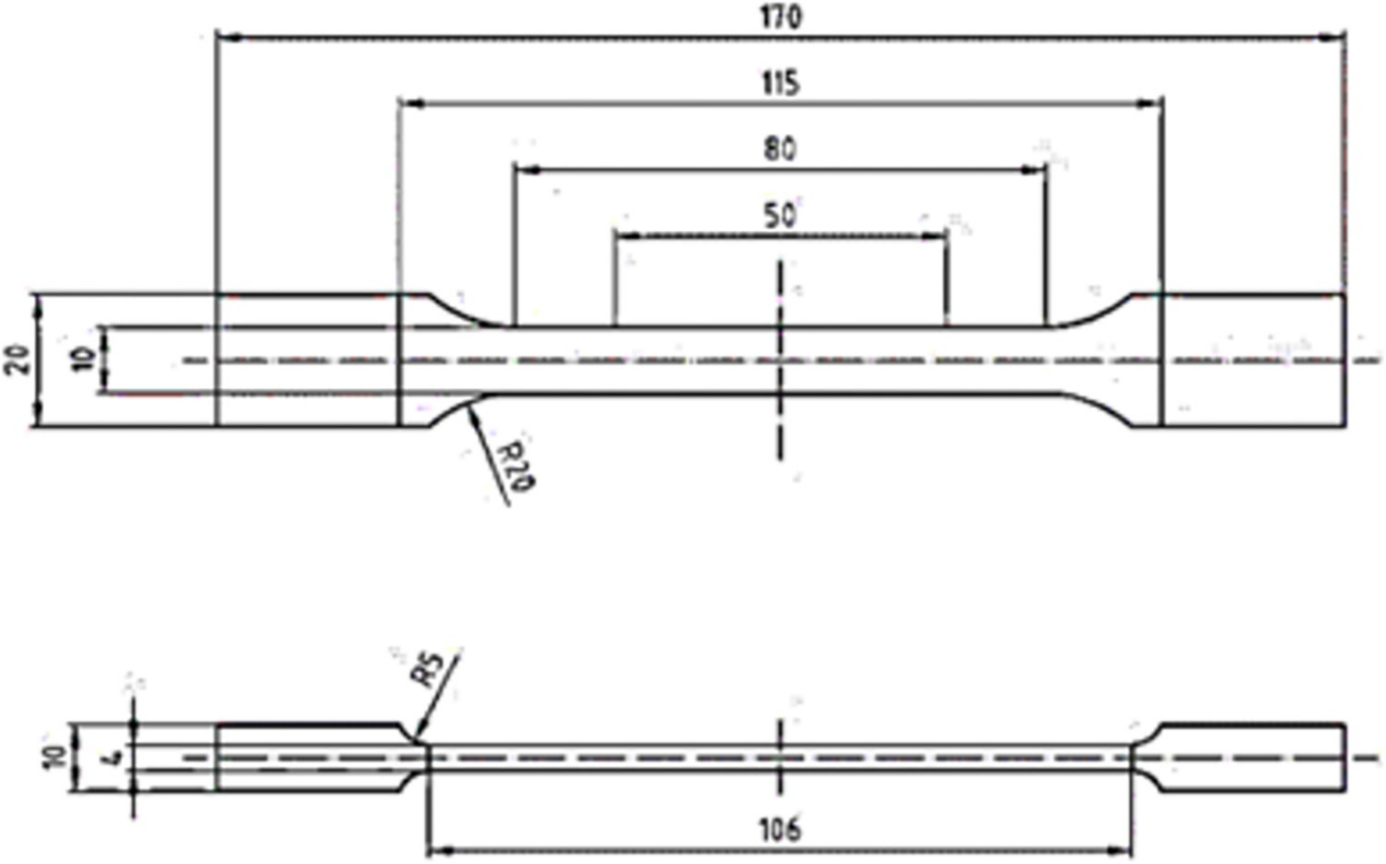
Dimensions (in millimeters) of the 3D-printed standard test specimens.

**Fig 2:**
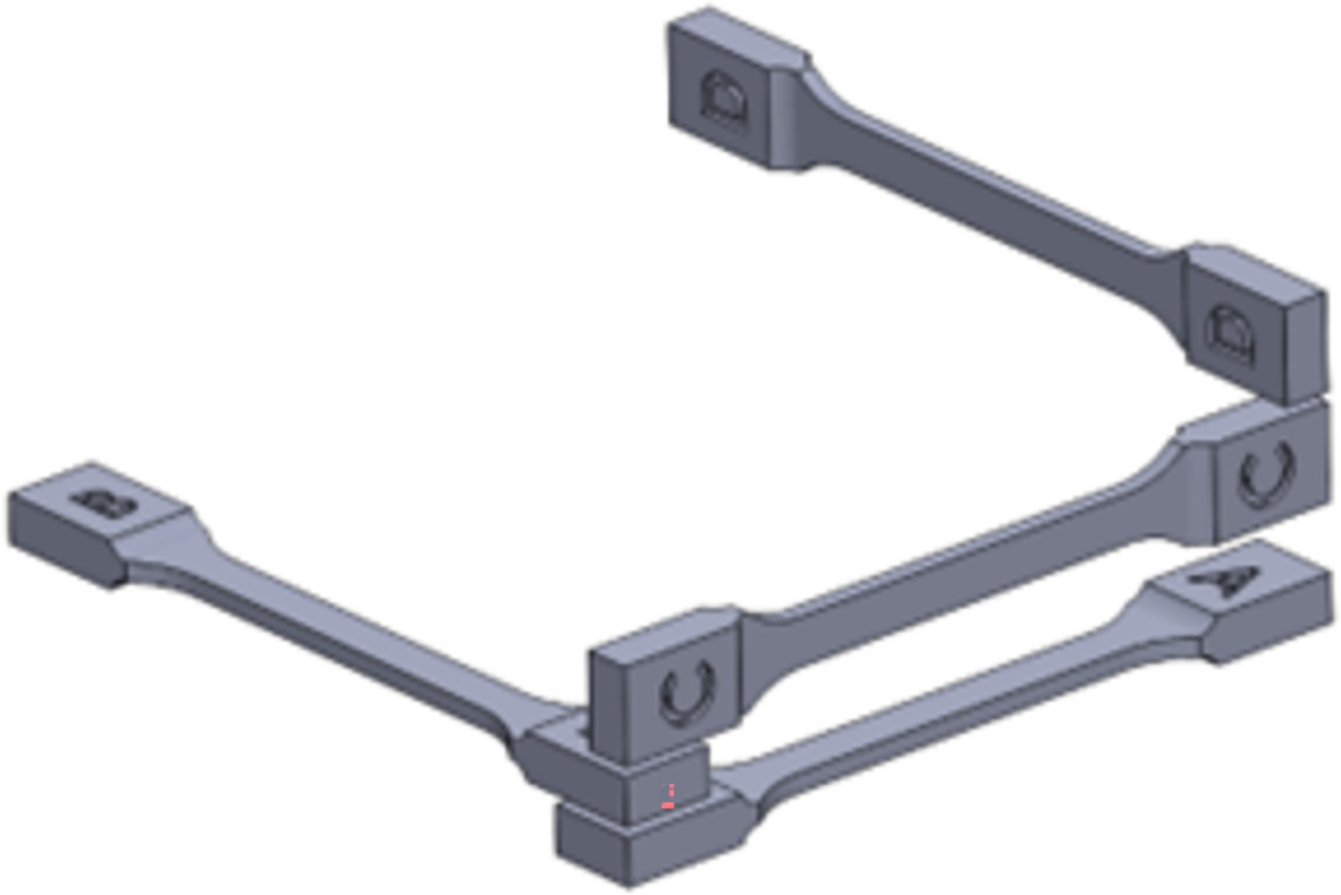
Different directions of the manufactured standard specimens.

Selective laser sintering machine FORMIGA P 100, equipped with 30 W CO_2_ was used to manufacture the studied specimens. The parameters of the printing process were the following: wavelength of laser 10.6 μm, scanning speed 5 m/s, positioning accuracy of laser beam ± 0.05 mm, hatching distance 0.25 mm, layer thickness 0.1 mm, layer thickness variation 0.01 mm, the melting temperature 172–180°C. The utilized PA2200 powder had the average grain size of 56 μm and was provided by EOS GmbH-Electro Optical Systems. In accordance with ISO-527 standard, dog-bone specimens with the dimensions shown in Fig 1 were produced for simple tension tests. To examine possible influences of build direction on the obtained responses, four different orientations for 3D printing of the specimens were selected. The letters A, B, C, and D in Fig 2 indicate these directions. The scanning direction of the 3D printer is parallel to the longitudinal axis of specimens A.

**Fig 3:**
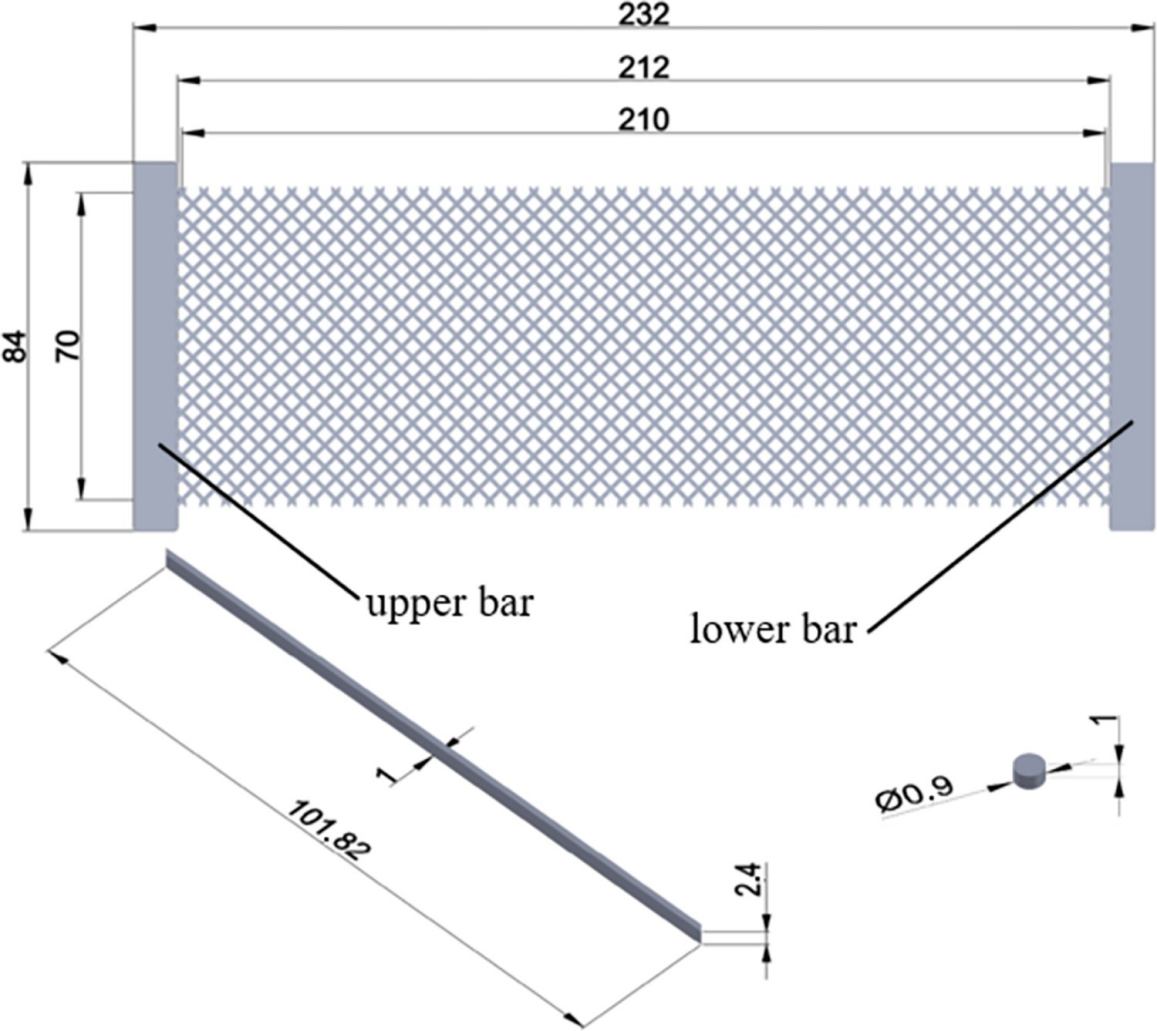
Geometry and dimensions (in millimeters) of the 3D-printed pantographic structure.

Details of the SLS-produced pantographic structures are brought in Fig 3. The fibers are interconnected by cylindrical pivots, each of which has the diameter and the height of 0.9 mm and 1 mm, respectively. The two parallel sets of the fibers are arranged perpendicularly, and each fiber is a prismatic beam with the shown dimensions for a complete fiber. The lattice structures were produced in direction A (according to Fig 2) to better control details of the pivots and placement of the structures inside the production chamber. To obtain tensile force-displacement responses of the whole structure, with the use of customized block inserts, the end plates are fixed in the grippers of the testing machine when conducting tensile loading experiments. The corresponding end plates to the upper and the lower jaws of the testing machine are shown in Fig 3.

## Experimental findings and discussion

**Fig 4:**
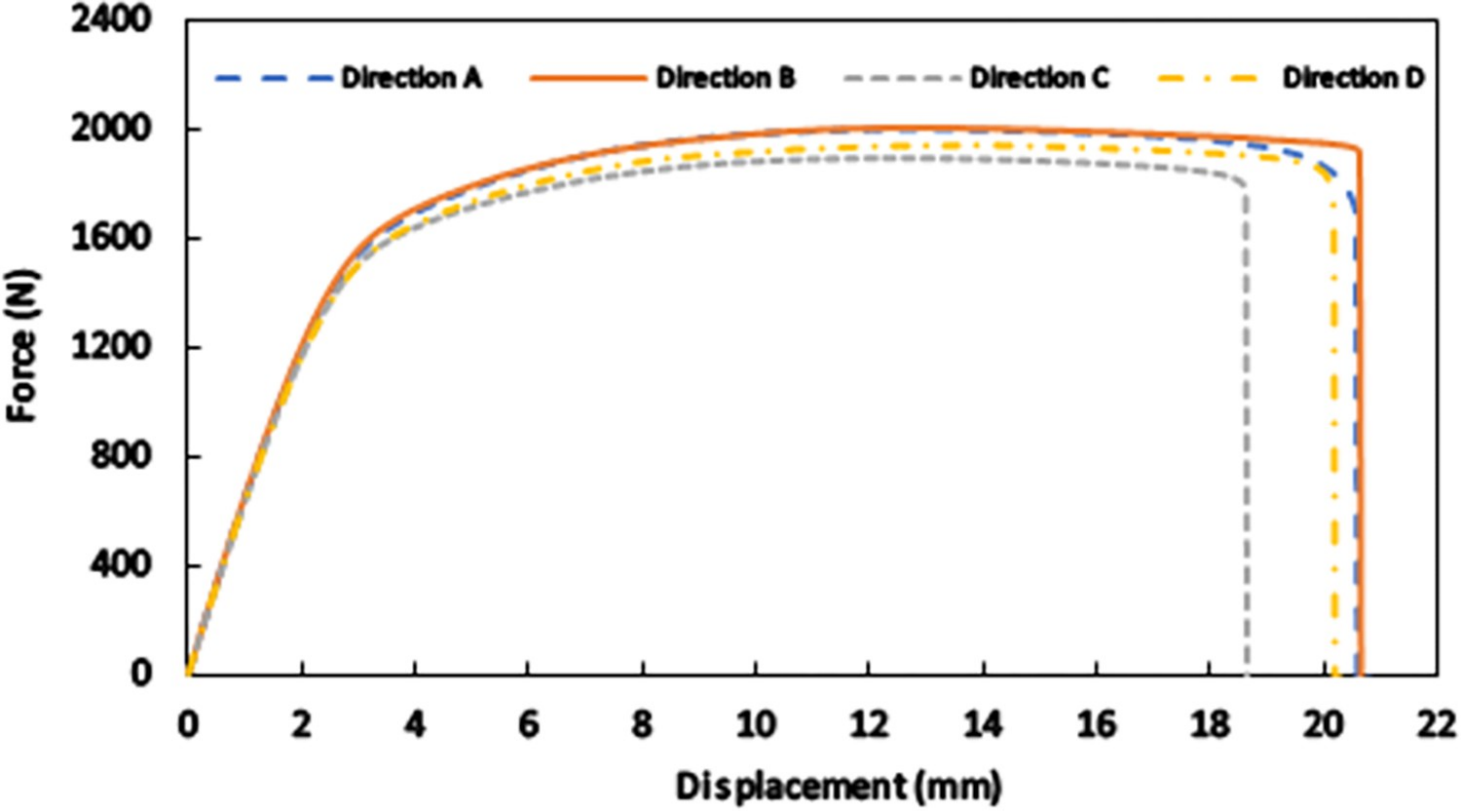
Tensile force-displacement response until fracture for standard specimens with different build orientations.

All the experiments on standard specimens and the pantographic structures were performed by an MTS Biosystem machine with the maximum load capacity of 25 kN. Each test was repeated three times to ensure the repeatability of the results. Simple tension tests under the loading speed of 0.05 mm/s were first conducted on standard specimens with different build orientations to obtain and compare the responses of all the examined directions. The achieved force-displacement curves until fracture are shown in Fig 4 in which, for a given amount of the applied displacement, a maximum difference of around 3.5% exists between the obtained forces for the four studied build orientations. In the elastic parts of the curves, very negligible differences much lower than those in the inelastic parts are seen, and this is in accordance with the latest findings reported in similar studies on possible anisotropies in selectively laser sintered polyamide 12 parts [17, 25, 33]. Accordingly, the results are practically assumed the same in this study, and the SLS-produced specimens are considered isotropic. It has been proved [34, 35] that possible anisotropy is significantly suppressed if, as is fulfilled in current work, recycled powders are not used in selective laser sintering. The force-displacement response shown in Fig 4 indicates that elastoplastic models [22, 23, 26] can address the observed behavior. More precisely, the elastic part is nonlinear and hyperelastic models are suitable to describe the elastic response of selectively laser sintered PA12 parts [21].

**Fig 5:**
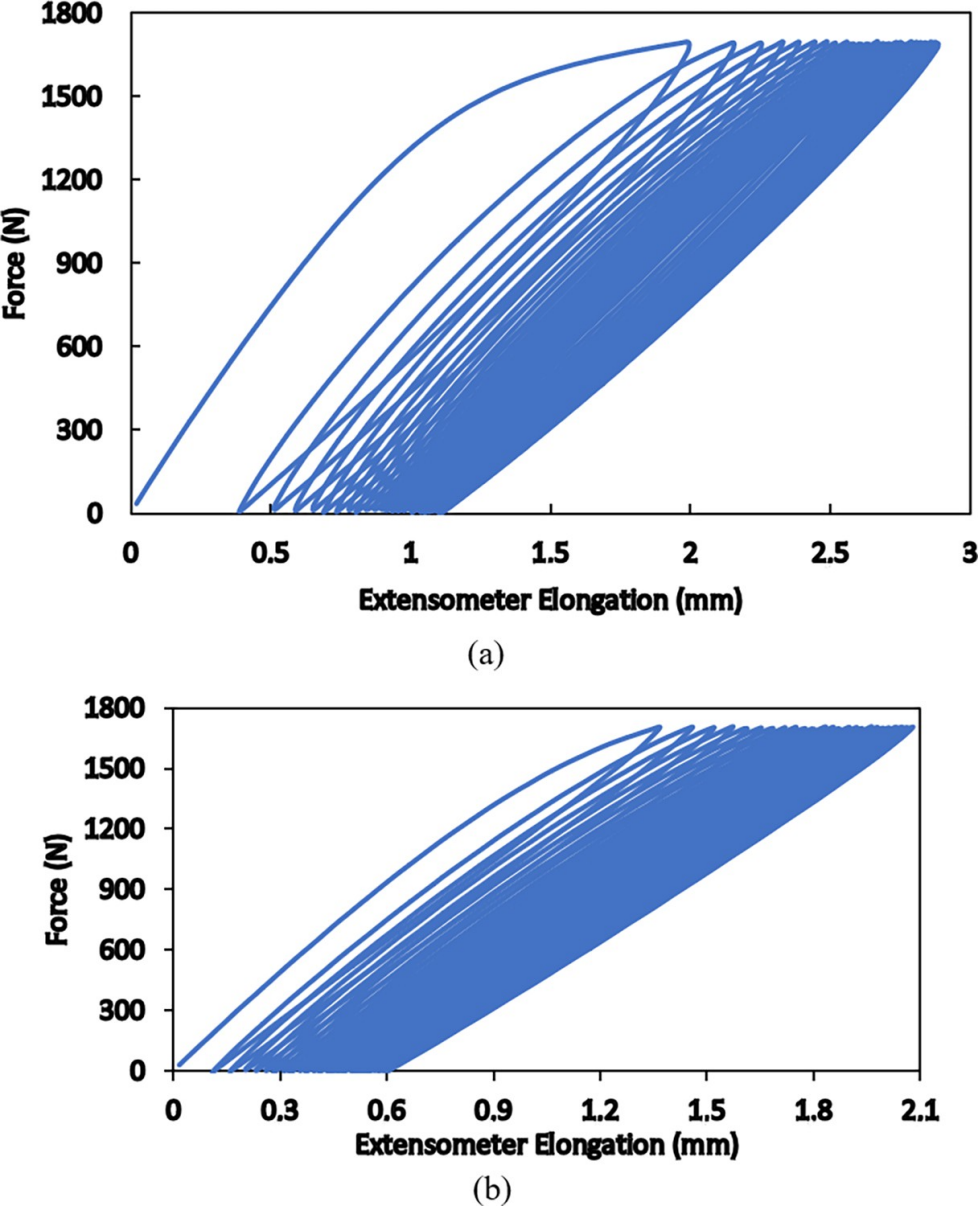
Cyclic responses of standard specimens at the loading speed of (a) 0.05 mm/s and (b) 0.5 mm/s for the maximum force of 1700 N.

**Fig 6:**
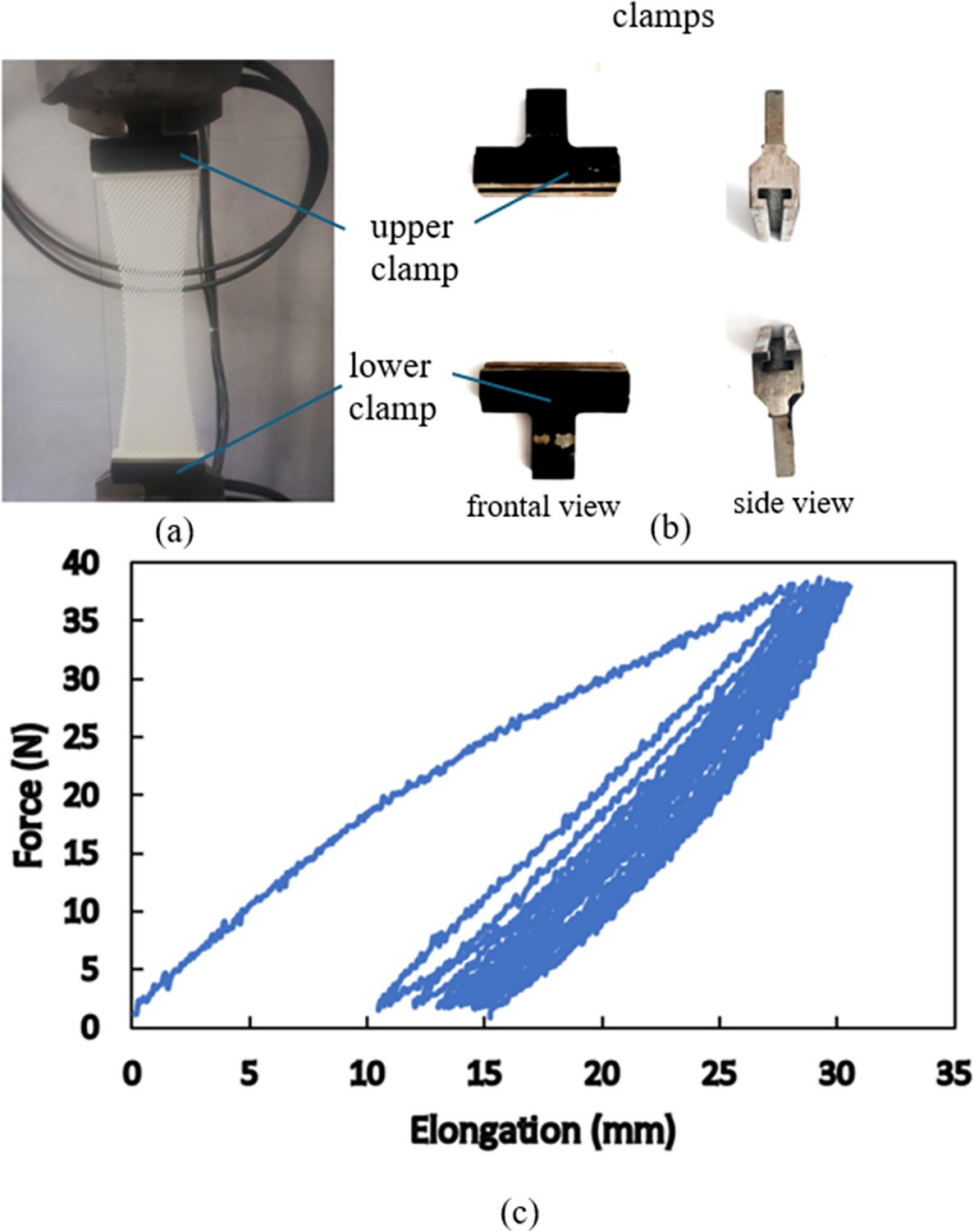
Cyclic tension test on the SLS-produced pantographic structure: (a) Deformed configuration of the structure under the application of maximum load, (b) details of the clamping, and (c) Force-displacement response under cyclic loading.

To understand more details of hyperelsaticity in SLS-produced specimens, cyclic loadings with different maximum loads and speeds are carried out on standard specimens. An extensometer is utilized to record elongation of the gauge length of 50 mm in the middle of each specimen. The force-elongation responses for the maximum load of 1700 N at two speeds of 0.05 mm/s and 0.5 mm/s for 25 loading/unloading cycles are shown in Fig 5(A) and 5(B). Since the maximum load is fixed, due to strain hardening of the material, elastic response appears upon the first unloading and in the subsequent loading/unloading cycles. The elastic response in nonlinear, however, and the cycle-by-cycle accumulation of residual strain accompanied by hysteresis is seen in the obtained results. Moreover, all details of the responses are rate dependent i.e., the material is more ductile, larger strains are achieved, and wider hysteresis appear in the cyclic response at a lower loading speed. These results are consistent with formerly reported findings [36, 37] and indicate that the viscous nature of the polymer is dominant in such conditions; thus, hyper-viscoelasticity governs the mechanical behaviors of selectively laser sintered polyamide 12. Similar responses with these details are obtained for different maximum loads beyond the elastic limit of the material and also for the produced pantographic structure, as is seen in Fig 6. In Fig 6(A), it is shown how deformations of different fibers accommodate uniaxial elongation of the whole structure.

**Fig 7:**
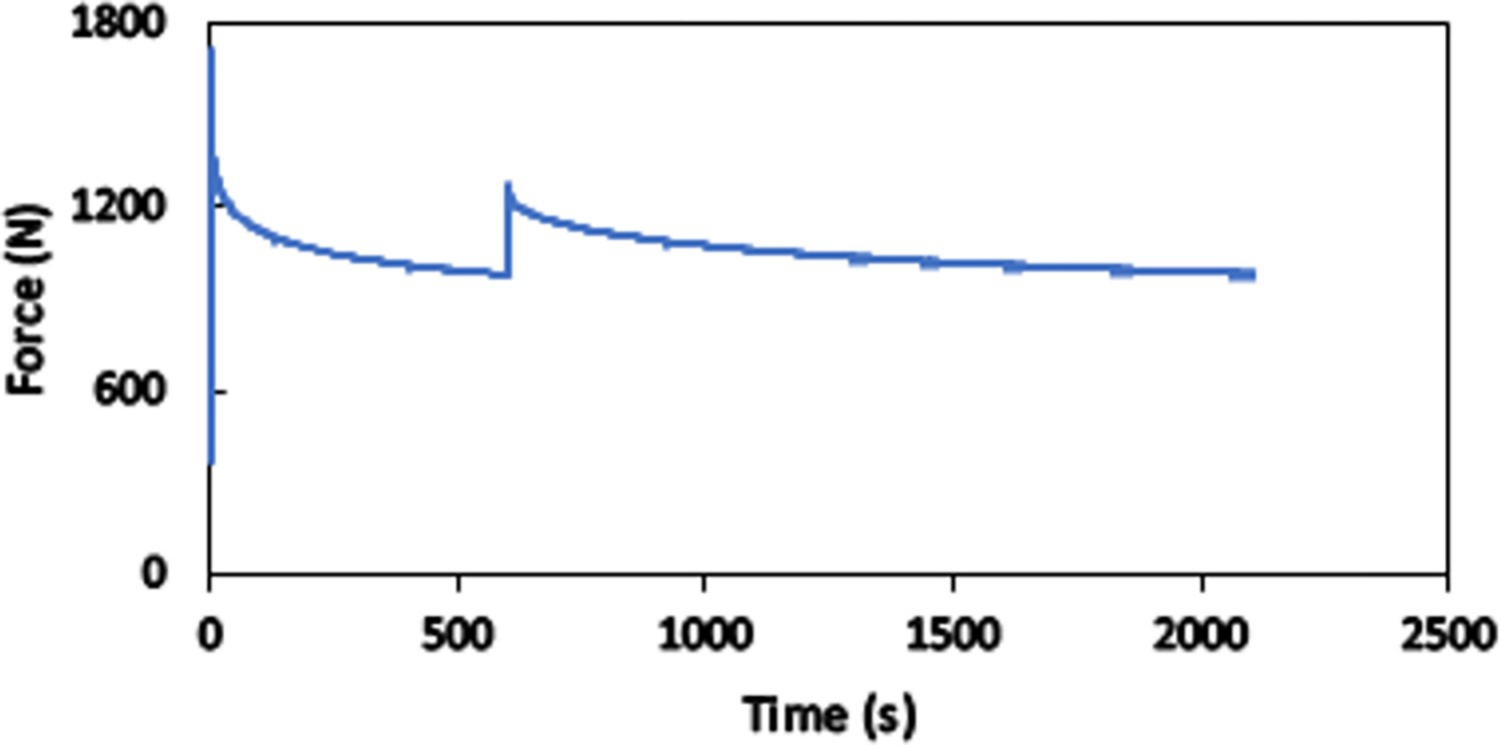
Results of two-step relaxation test on a standard specimen.

In order to assess viscoelasticity in the responses of the products, a two-stage relaxation test is performed. A total displacement of 3 mm is first rapidly applied to the whole length of a specimen, and it is kept fixed for 10 minutes. Then, the displacement is increased to 3.5 mm and is held fixed in the rest of the experiment. Variations in the applied force to the specimen with time are shown in Fig 7. The drastic decrease in the amount of force in each stage clarifies the presence of viscose response in the material.

**Fig 8:**
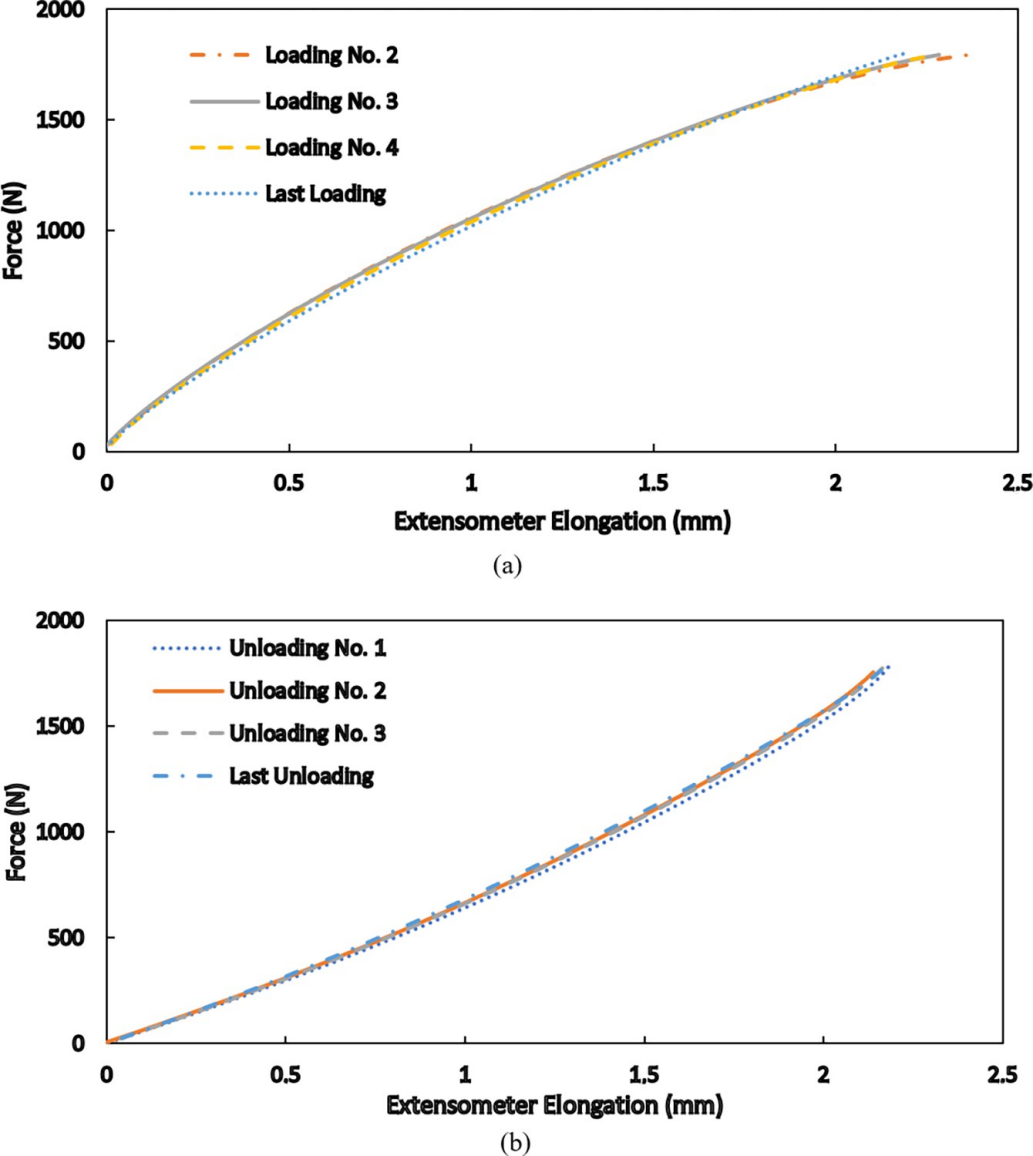
Comparison of selected force-elongation responses during (a) loading and (b) unloading cycles by shifting the curves to the origin of the graph.

To reveal more characteristics of the products’ behavior, for the maximum applied force of 1800 N, the response of the second loading cycle is plotted from the origin of the graph. By shifting the curves to the zero point, the responses of the third, the fourth, and the last loading cycles are also plotted to compare the responses. As is seen in Fig 8(A), the curves are nearly identical and resemble the force-elongation response of a hyperelastic material. Similarly, by shifting the residual strains to zero, the unloading curves of the first three unloading cycles, as well as the last unloading, are plotted in Fig 8(B). These curves are also very close to each other, and the shown comparisons indicate that a hyperelastic model accompanied by generalizations to include hysteresis and the accumulation of residual strain is able to predict the observed results. This model is developed in the next section.

## Constitutive model

A hyper-viscoelastic model is developed in this section to predict the mechanical behaviors of selectively laser sintered PA12 products. Founded on the works of Goh et al., [38] and Pawlikowski [39, 40], to address the experimentally observed responses in this research, instantaneous stress is stated as a convolution integral of a strain-dependent function *σ*_0_(*ε*) and a time-dependent function *g*(*t*):

$$\sigma (\varepsilon ,t)=\int_{0}^{t}g\left(t-s\right)\frac{d\sigma_{0}(\varepsilon )}{ds}ds$$ (1)

where *s* represents a historical time variable. The function *g*(*t*) may be defined by means of a Prony series as:

$$g(t)=g_{\infty }+\sum_{i=1}^{N}g_{i}∙e^{-\frac{t}{\tau_{i}}}$$ (2)

in which *g*_∞_ and *g*_*i*_ are dimensionless characteristic amplitudes and *g*(0) = 1; thus, $g_{\infty }=1-\sum_{i=1}^{N}g_{i}$.

Consequently, the function *σ*_0_(*ε*) represents the instantaneous stress-strain relationship as *g*(0) = 1 and *σ*(*ε*,0) = *σ*_0_(*ε*). Also, *g*_∞_*σ*_0_(*ε*) is the long-term or equilibrium stress-strain relationship since *σ*(*ε*,∞) = *g*_∞_*σ*_0_(*ε*). The parameters *τ*_*i*_ indicate the relaxation times; thus, Eq (1) provides a general equation for nonlinear visco-elasto-plasticity using which the so-called fading memory can be incorporated into any constitutive model. Based on relationships (1) and (2), the following discretized equation for a limited number of time intervals can be obtained:

$$\sigma (t)=g_{\infty }\sigma_{0}(t)+\sum_{i=1}^{N}\int_{0}^{t}g_{i}exp(-\frac{t-s}{\tau_{i}})\frac{d\sigma_{0}(s)}{ds}ds$$ (3)

Using this relationship, the stress function is split into a long-term response and a viscoelastic contribution. Eq 3 can be written as

$$\sigma (t)=g_{\infty }\sigma_{0}(t)+\sum_{i=1}^{N}h_{i}(t)$$ (4)

where the stress term *h*_*i*_(*t*) is:

$$h_{i}(t)=\int_{0}^{t}g_{i}exp(-\frac{t-s}{\tau_{i}})\frac{d\sigma_{0}(s)}{ds}ds$$ (5)

Based on the numerical algorithm presented by Goh et al., [38], for a time interval (*t*_*n*_, *t*_*n*+1_) with the time step of Δ*t* = *t*_*n*+1_−*t*_*n*_, one can write:

$$h_{i}(t_{n+1})=exp\left(-\frac{\Delta t}{\tau_{i}}\right)h_{i}(t_{n})+g_{i}\int_{t_{n}}^{t_{n+1}}g_{i}exp(-\frac{t_{n+1}-s}{\tau_{i}})\frac{d\sigma_{0}(s)}{ds}ds$$ (6)

As a result, the following recursive formula is obtained for updating the stress *σ*(*t*_*n*+1_):

$$\sigma (t_{n+1})=g_{\infty }\sigma_{0}(t_{n+1})+\sum_{i=1}^{N}(exp(-\frac{\Delta t}{\tau_{i}})h_{i}(t_{n})+g_{i}\frac{1-exp(-\frac{\Delta t}{\tau_{i}})}{\frac{\Delta t}{\tau_{i}}}\left(\sigma_{0}(t_{n+1})-\sigma_{0}(t_{n})\right)).$$ (7)

The strain dependent function *σ*_0_(*ε*), which is referred to as the core model in this work, is selected based on the experimental findings discussed in the previous section. Accordingly, Mooney-Rivlin hyperelastic models are considered suitable, and a two-parameter potential function is implemented here:

$$\Psi =c_{10}(I_{1}-3)+c_{01}(I_{2}-3)$$ (8)

where *I*_1_, *I*_2_ are the first and the second invariant of the right Cauchy stress tensor, respectively, and the two constants *c*_10_ and *c*_01_ are material parameters. Consequently, as a function of stretch ratio λ along the loading direction, the core model is [21]:

$$\sigma_{0}=\frac{2}{\lambda^{4}}(\lambda^{3}-1)(c_{01}+c_{10}\lambda )$$ (9)

With the use of Eq 9 in formulation (7), a hyper-viscoelastic model is obtained that is inherently capable of predicting rate dependency as well.

**Fig 9:**
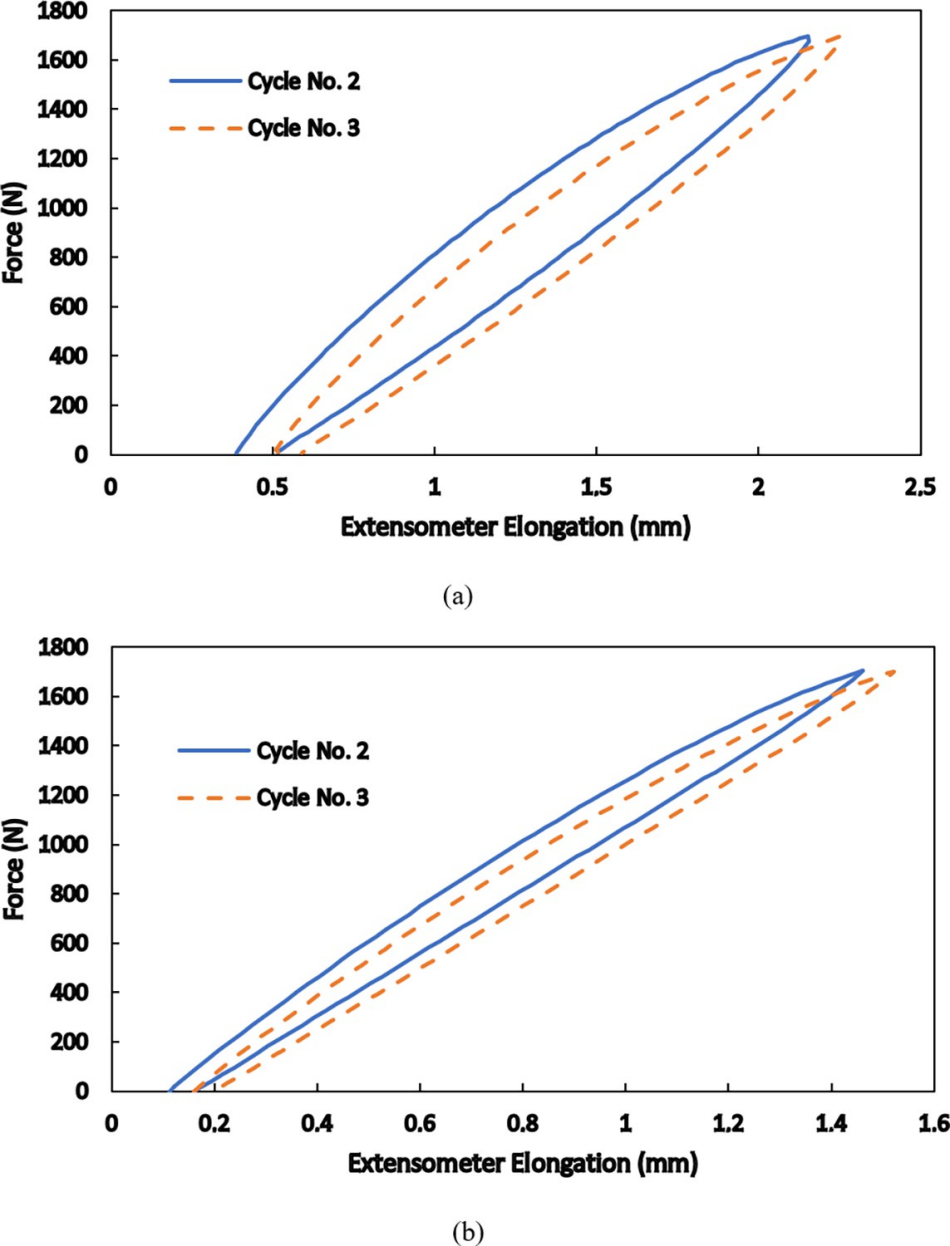
The utilized responses of the second and the third cycles under the maximum force of 1700 N at the loading speed of (a) 0.05 mm/s and (b) 0.5 mm/s for identification of the material parameters.

Determination of the number *N* of relaxation times *τ*_*i*_ was performed based on one relaxation test. The number of relaxation times *N* was determined by means of the algorithm presented in [38]. It was incorporated in the fitting of the constitutive model to the relaxation curve (Fig 7). To calibrate the rest of the required material parameters with the use of a wide range of data, results of the second and the third loading/uploading cycles of the tension tests under the maximum force of 1700 N at two loading speeds of 0.05 mm/s and 0.5 mm/s were simultaneously utilized (Fig 9A and 9B). The parameters identification was conducted by means of curve-fitting procedure using the Levenberg-Marquardt algorithm [40].

## Numerical results

According to the aforementioned procedure, we obtained the number of relaxation times *N* = 15 together with the material parameters provided in Table 1. To evaluate the validity of the determined constants, equivalent linear elastic response of the material is compared with available data in the literature. Based on the 2-parameter Mooney-Rivlin model, the modulus of rigidity is 2(*c*_10_+*c*_01_).

**Table 1:** Values of the identified constants for the SLS-produced parts.

*N*	*g*_1_	*g*_2_	*g*_3_	*g*_4_	*g*_5_	*g*_6_	*g*_7_	*g*_8_
15	0.0246	0.0451	0.012	0.0453	0.0161	0.0168	0.0507	0.011
*g*_9_	*g*_10_	*g*_11_	*g*_12_	*g*_13_	*g*_14_	*g*_15_	*c*_01_ (MPa)	*c*_10_ (MPa)
0.11	0.0392	0.0168	0.0161	0.0379	0.128	0.0239	-78.64	388.74
*τ*_1_ (s)	*τ*_2_ (s)	*τ*_3_ (s)	*τ*_4_ (s)	*τ*_5_ (s)	*τ*_6_ (s)	*τ*_7_ (s)	*τ*_8_ (s)	*τ*_9_ (s)
0.0234	0.1	0.34	0.43	0.53	1.447	1.817	2.282	7.746
*τ*_10_ (s)	*τ*_11_ (s)	*τ*_12_ (s)	*τ*_13_ (s)	*τ*_14_ (s)	*τ*_15_ (s)			
33.02	58.67	79.21	140.75	600	2557.6			

This leads to the Young’s modulus of around 1.86 GPa for the present products. This number is in a good agreement with the reported elastic modulus of polyamide 12 produced by selective laser sintering‎ [21, 41] and indicates that the proposed hyper-viscoelastic model in the present work predicts the elastic response of SLS-produced PA12 with a reasonable accuracy.

**Fig 10:**
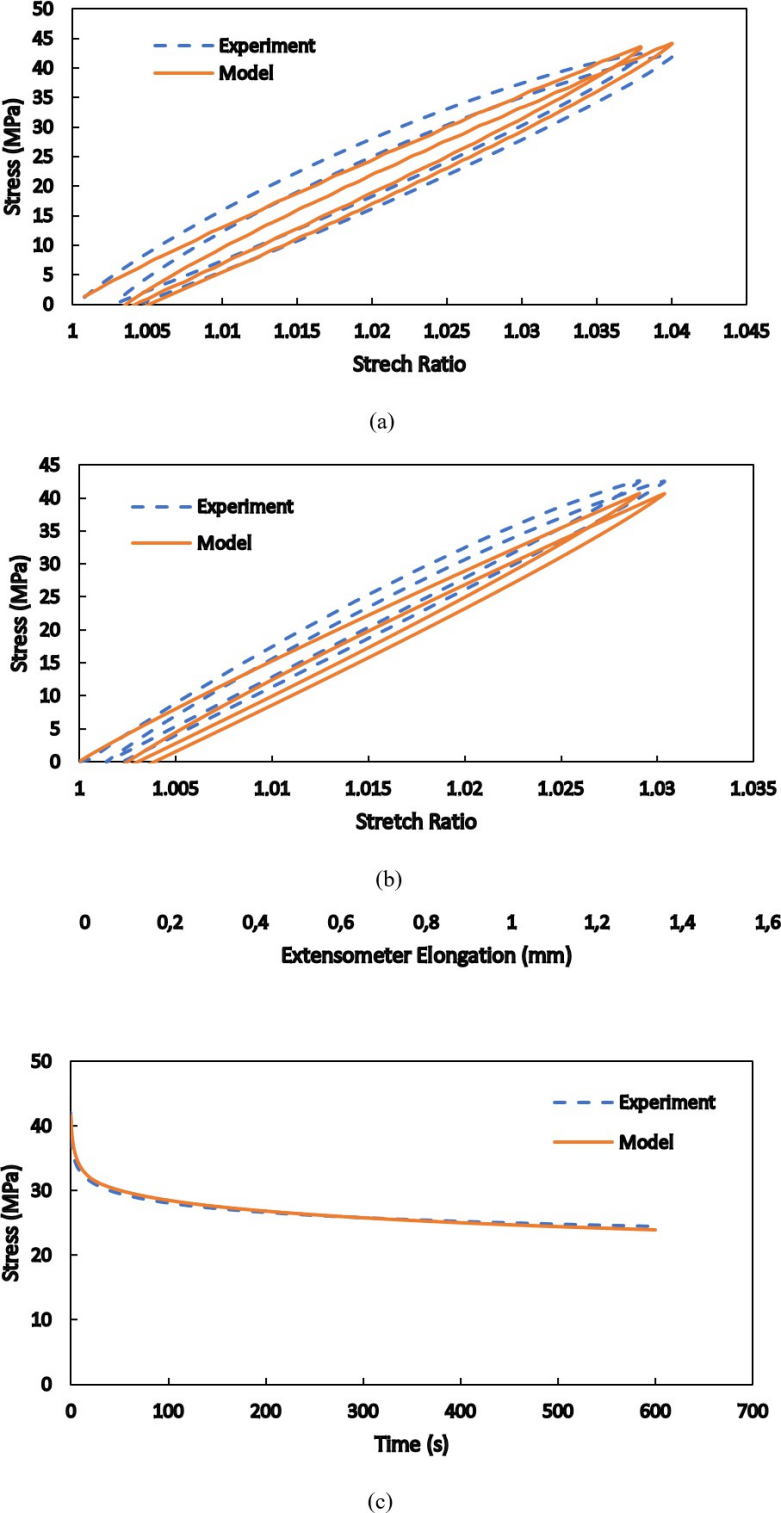
Numerical reproduction of the empirical responses utilized for identification of the material parameters: (a) stress-stretch ratio at the loading speed of 0.05 mm/s, (b) stress-stretch ratio at the loading speed of 0.5 mm/s, and (c) relaxation for the fixed total elongation of 3 mm.

**Fig 11:**
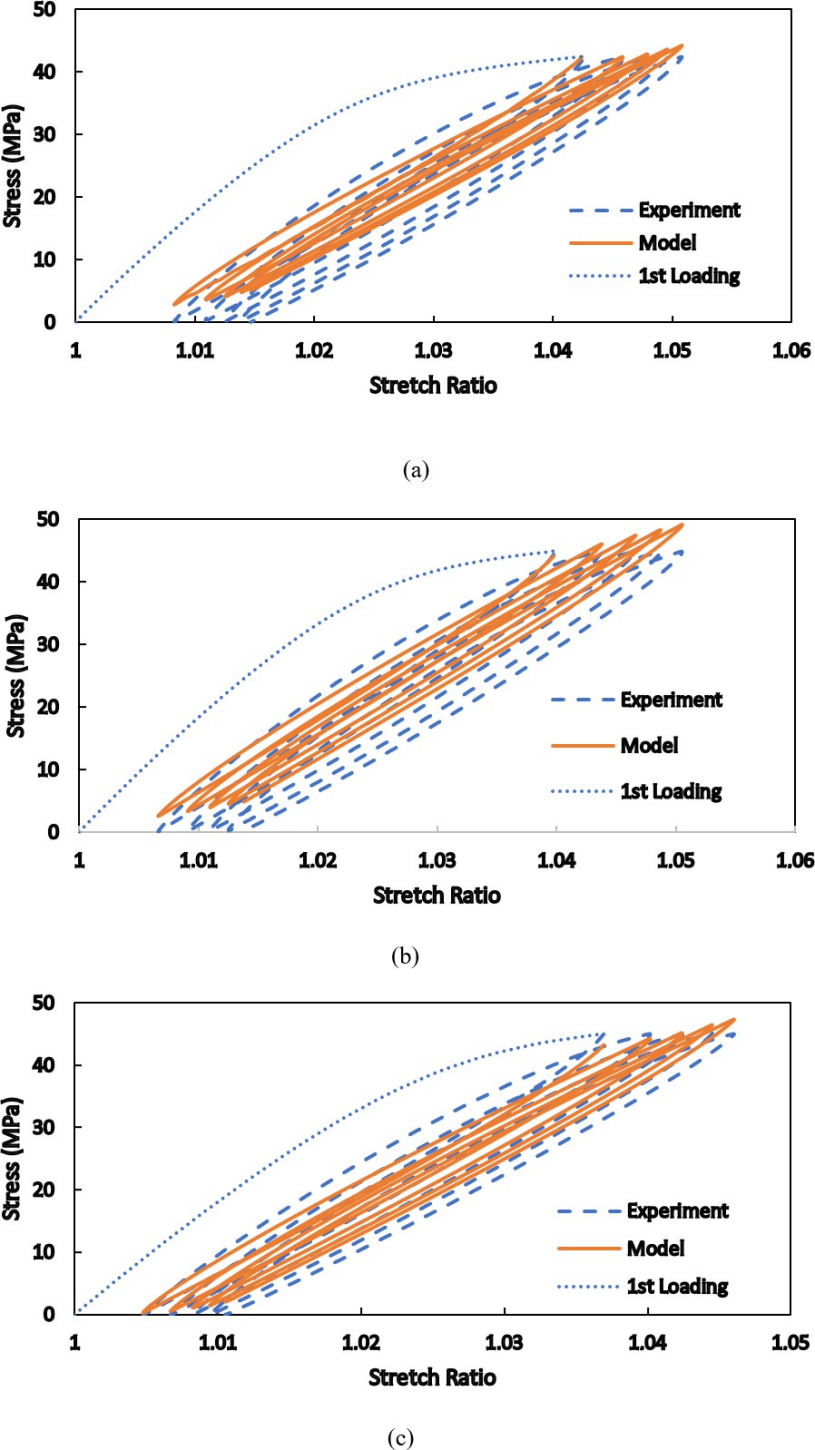
Comparison between the numerical and experimental results for the loading speed of (a) 0.05 mm/s, (b) 0.2 mm/s, and (c) 0.5 mm/s.

Using the determined parameters, the numerical responses shown in Fig 10 are obtained. As is seen, the experimental curves are reproduced with an acceptable accuracy. For further evaluation of the validity of the proposed model in different conditions, as well as the robustness of the implemented algorithm to evaluate the material parameters, cyclic tensile loadings with the maximum force of 1800 N at three different loading speeds are investigated in Fig 11. The first unloading cycle, where just hyper-viscoelasticity begins to appear, followed by four subsequent loading/unloading cycles are numerically simulated, and the results are compared with the experimental responses. In the curves of the three studied loading speeds of 0.05 mm/s, 0.2 mm/s, and 0.5 mm/s, a maximum difference of 9.9% exists between the theoretical and the corresponding empirical results. The calibration of the model parameters and the model validation was conducted at those particular loading speeds to be consistent with the previous works [9, 42] where different approach of modelling structures made of PA12 is presented and with the study [21] where hyperelastic modelling of PA12 is shown.

**Fig 12:**
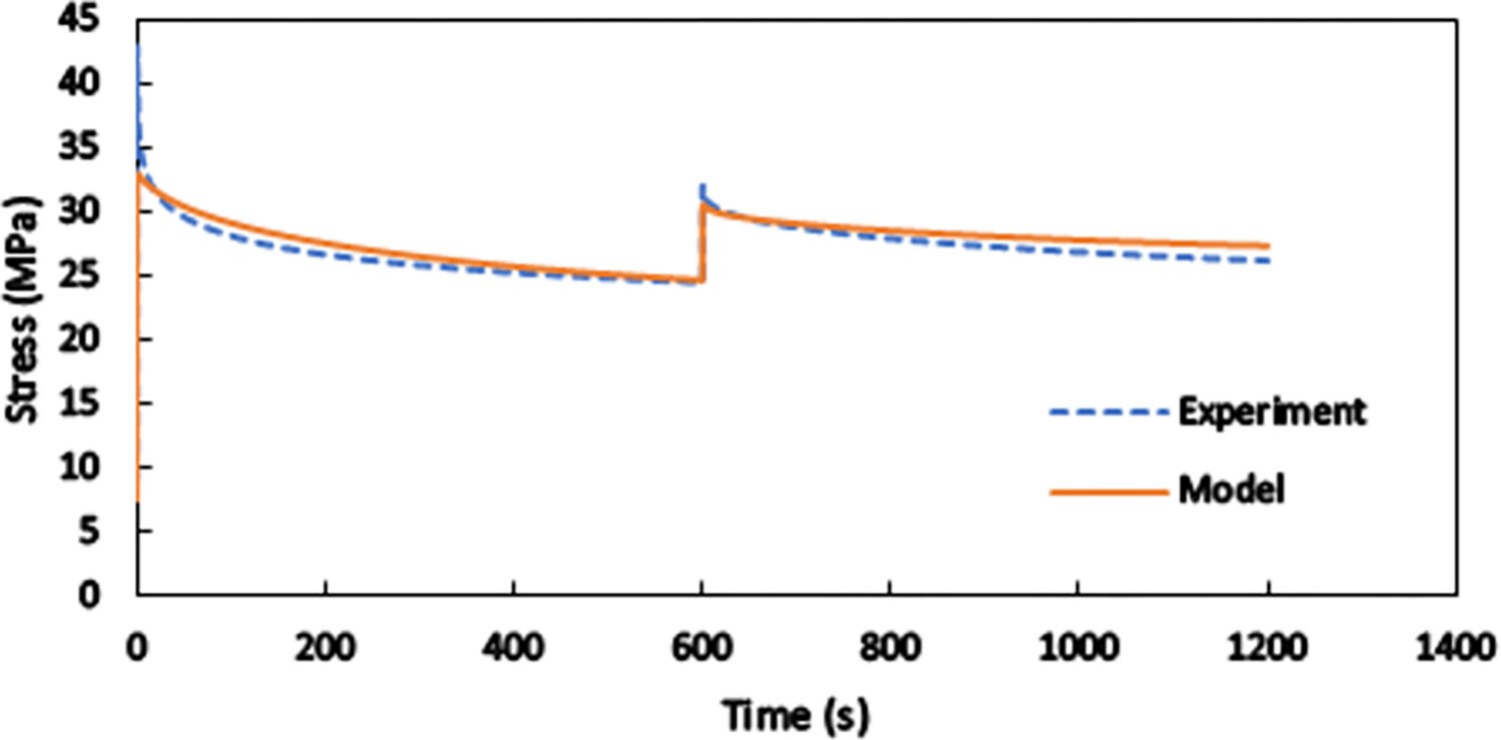
Comparison between the numerical and experimental results for two-step relaxation test.

A two-step relaxation test, where the total elongations of 2 mm and 2.5 mm are sequentially applied to the whole length of a standard specimen, is investigated in Fig 12. Each step lasts for 10 minutes, and variations of the required force with time are determined both numerically and experimentally. In this condition, the viscous nature of the material is considerably more pronounced than the hyperelastic response, which is more dominant in short-term loadings. The brought responses and comparisons in Figs 11 and 12 indicate that the presented model is inherently able to consider rate dependency in the mechanical behaviors of SLS-produced PA12 and that the numerical results are in a good agreement with the experimental ones for various types of short-term and long-term loadings.

**Fig 13:**
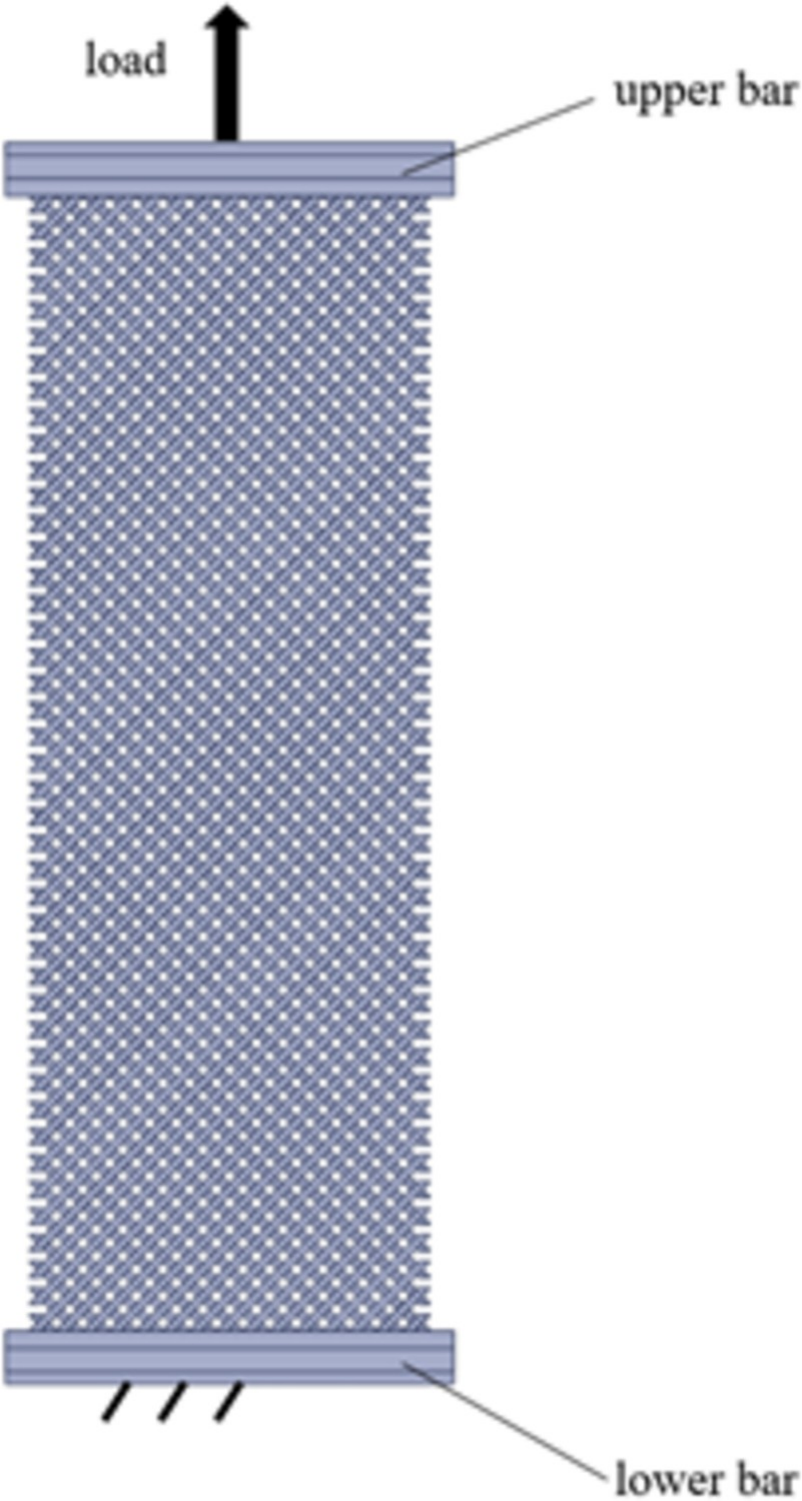
Boundary conditions in the finite element model of the studied pantographic structure.

**Fig 14:**
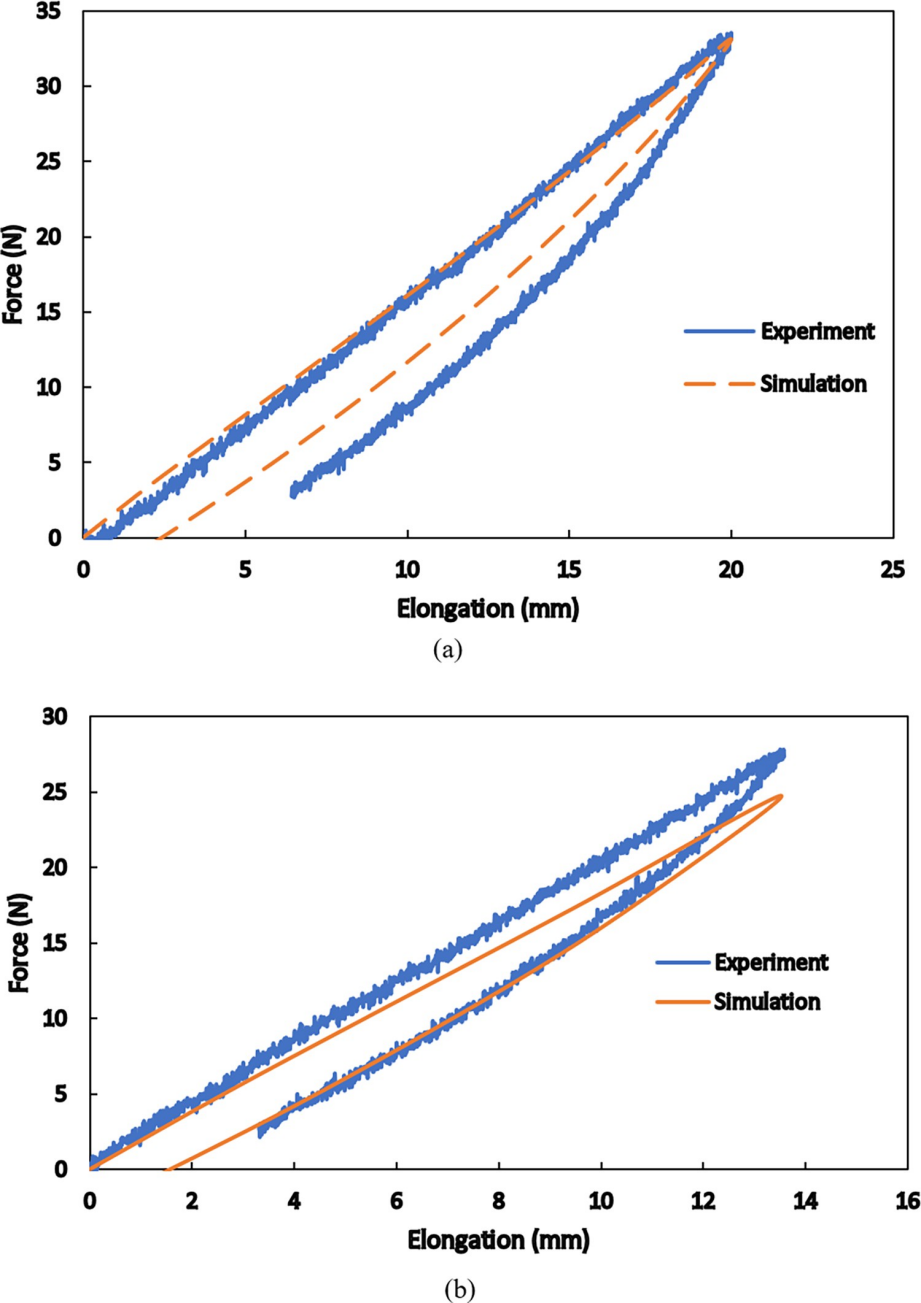
Comparison between the numerical and experimental results for (a) the first and (b) the second loading-unloading cycle on the SLS-produced pantographic structures.

Once the base material is characterized and its constitutive model is derived, the mechanical behaviors of any polyamide 12 product fabricated by selective laser sintering can be predicted for practical purposes. As a case study, a pantographic structure under the application of two successive tensile loading-unloading cycles is simulated by finite element method, and the theoretical and empirical force-displacement responses are compared to each other. Finite element simulations are conducted in software ANSYS for the geometry shown in Fig 3. Three-dimensional tetrahedral elements are used to mesh the structure. The lower surface of the lower bar is fixed so that all the degrees of freedom are blocked. The load is applied on the upper surface of the upper bar of the structure. These boundary conditions are depicted in Fig 13. The formulated hyper-viscoelastic constitutive model is implemented by defining a new material model consisting of 2-parameter Mooney-Rivlin hyperelastic model and the Prony shear relaxation module. To complete the new material definition, the identified values of the parameters are utilized in the software. The force-displacement curves of the first and the second loading-unloading cycles are separately shown in Fig 14A and 14B, respectively. In the first cycle, the structure is stretched by 20 mm within 1200 s and then is unloaded at the same rate. Then, in the second cycle, 13.5 mm extension within 820 s is applied to the structure followed by unloading at the same rate. The maximum difference of nearly 10.7% is observed between the numerical and the empirical results at the end of the second loading cycle. The differences between the model and the experimental findings are due to the fact that our model covers a relatively wide range of strain rate deformation of the polyamide. Calibration of the model parameters for only one strain rate would result in a very good fit of the model to the experimental curves, however, such a model could have been used only in the case of the material deformation with that particular strain rate. Since this would have been a limitation of the PA12 modelling, we decided to formulate a more universal constitutive model to be able to simulate deformation of PA12 with various strain rates. However, the results indicate the reliability of the proposed model in studying the behaviors of SLS-produced PA12 parts with complex geometries.

## Conclusions

The experimental and theoretical investigations in the present work reveal hyper-viscoelasticity of SLS-produced PA12 parts. Inelastic loading is first induced in the examined products, and the material’s responses in the unloading cycle followed by cyclic loading-unloading until the previously-applied maximum load are studied. Different maximum loads and strain rates are considered, and it is observed that the material exhibits a same hyperelastic response that is shifted cycle by cycle during the cyclic loadings; however, the *core* hyperelastic response differs from loading to unloading cycles so that hysteresis exists in the strain-strain curves of the material. Stress relaxation experiments are also conducted, and these short-term and long-term findings are utilized in deriving the presented hyper-viscoelastic model. In the proposed approach, 2-parameter Mooney-Rivlin hyperelastic model and the Prony shear relaxation module are employed as the strain-dependent and time-dependent functions using which a convolution integral is developed to derive the constitutive equations. This model is inherently able to consider rate dependency in the mechanical behaviors of SLS-produced PA12 parts. The numerically-predicted results are found to be in a good agreement with the experimental ones for various types of short-term and long-term loadings, and this indicates the capability of the proposed model in studying any product with general geometries. To approve this ability, typical pantographic structures are manufactured with the same production settings as those applied in 3D printing of the studied standard tensile specimens. Finite element implementation of the constitutive equations is performed in software ANSYS, and the produced pantographic structure is simulated under uniaxial tension. The numerical results coincide with the empirical findings for tensile loading of the 3D-printed specimen with a reasonable accuracy. The presented model gives the equivalent elastic modulus of nearly 1.86 GPa for the products that is very close to the formerly-reported number in the literature for polyamide 12 produced by selective laser sintering‎. Any stress-strain function is applicable as the core model of the presented formulation so this approach in a general method in deriving rate-dependent hyper-viscoelastic constitutive equations any material and production scheme.

## Supporting information

S1 File Figs 10–12. The file, which is in the format.xlsx, comprises data and graphs presented in Fig 10A and 10B (tab “Identification 2nd&3rd cycle”), Fig 10C (tab “Stress relaxation”) Figs 11 and 12 (tab “Validation”).
(XLSX)
